# New Agents Targeting Angiogenesis in Glioblastoma

**DOI:** 10.1155/2011/878912

**Published:** 2011-11-02

**Authors:** Eleni Timotheadou

**Affiliations:** Department of Medical Oncology, Aristotle University of Thessaloniki School of Medicine and General Hospital “Papageorgiou”, 54303 Thessaloniki, Greece

## Abstract

Glioblastoma is the most common malignant glioma in adults, and despite recent advances in standard treatment, the prognosis still remains dismal, with a median survival of 15 months. The incorporation of bevacizumab in the standard treatment of relapsed glioblastoma has been a significant step towards combining targeted agents with chemotherapy, and there is an increasing number of new antiangiogenic agents in various stages of development, that are being tested both in relapsed and newly diagnosed disease, alone or in combination with standard treatment. The relatively favorable toxicity profile for most of them presents an advantage, but several concerns arise regarding their actual efficacy on the clinical level and the most efficient schedule of administration for each of them, as their molecular targets and patterns of action may vary significantly. This may lead to future modifications of the current rational of administering these agents concomitantly with initial chemotherapy or maintenance treatment.

## 1. Introduction

Glioblastoma is the most common malignant glioma in adults and, in spite of its relatively low incidence in the general population, it is a disease with extremely high morbidity and mortality. Practically all patients eventually die of a disease-related complication. The current standard of care includes maximum surgical resection and radiotherapy with concomitant temozolomide followed by adjuvant temozolomide as initially described by Stupp et al. in 2005 [1]. Despite the improvement in survival observed in this trial, the majority of patients survive less than 2 years from diagnosis, while less than 5% will be alive in 5 years, experiencing significant deterioration in their quality of life and multiple debilitating symptoms. Therefore, the demand for more effective treatment remains imperative and the introduction of new agents in clinical trials continuously produces new data that will hopefully lead to better treatment results in the near future.

This short paper will focus on bevacizumab and the novel antiangiogenic agents cediranib, cilengitide, sunitinib, sorafenib, vandetanib, aflibercept, ABT-510 (thrombospondin-1) XL184, and tandutinib that are currently in various stages of clinical development both on recurrent and untreated glioblastoma.

## 2. Targeting Angiogenesis in Glioblastoma

Inhibition of angiogenesis has been a long-standing therapeutic target, as gliomas are highly vascular tumors and several researchers have demonstrated the significance of the vascular endothelial growth factor (VEGF) family and its receptors in the angiogenesis and proliferation of the glioma cells [2, 3]. The rapid growth that characterizes gliomas results to regional hypoxia, which stimulates VEGF secretion via the HIF-1a activation [4]. VEGF-A binds to VEGFR-2 in the vascular wall and promotes endothelial cell migration and proliferation, leading to formation of new vessels and recruitment of bone marrow-derived endothelial precursor cells in the circulation, that are directly incorporated into the tumor vessels [5, 6]. These vessels have several abnormal features as large diameter, tortuous route, decreased pericyte coverage, and increased thickness of basement membrane. The blood flow and permeability are increased, and the transport properties are altered. The tumor environment develops areas of hypoxia, increased interstitial pressure, and necrosis, features that are diagnostic marks of glioblastoma. The change of permeability affects the blood-brain barrier and contributes to the vasogenic cerebral edema formation that is usually present on diagnosis [7]. 

Angiogenesis is a complex, interactive procedure involving several molecular pathways. VEGF plays a key role and besides hypoxia it can be activated by many other factors as acidosis, oncogenes and tumor suppressor genes (kit and p53), cytokines [8] (EGF, FGF-b, PDGF), and signal transduction pathways as the PI3k/Akt and Ras/MAPK. Also, various molecules can activate angiogenesis under certain conditions, like the angiopoietin-1/angiopoietin-2/tie-2 signaling pathway, the insulin-like growth factor, hepatocyte growth factor, interleukins, tumor necrosis factor-*α*, and the cyclooxygenase-2 [9, 10]. Cell surface integrins *ανβ*3 and *ανβ*5, matrix metalloproteinase (MMP)-2 and MMP-9, and tenascin-C also promote angiogenesis via regulation of endothelial cell migration and invasion [11, 12]. It has been previously shown that the Notch signaling pathway has a critical role in vascular development [13]. Dll-4, a membrane-bound ligand for Notch-1 and Notch-4 is selectively expressed in some tumor endothelia, is induced by VEGF and hypoxia and has been considered as a potential therapeutic target [14]. 

However, the extensive preclinical studies have proved inadequate to fully elucidate the mechanisms of action of the anti-VEGF agents. Besides induction of endothelial cell apoptosis and inhibition of new vessel formation, it is also hypothesized that anti-VEGF agents may “normalize” the tumor vessels by decreasing permeability and interstitial pressure and thus reducing hypoxia and improving delivery of cytotoxics, when given in combination [15, 16]. There is clinical evidence of such normalization in patients who were treated with bevacizumab for colorectal cancer or recurrent glioblastoma and in recurrent glioblastoma patients treated with cediranib, a novel pan-VEGF inhibitor (AZD2171, Recentin) [17–19].

## 3. Bevacizumab

Bevacizumab (Avastin) has been the first antiangiogenic agent to gain approval for administration in recurrent glioma by FDA in May 2009. This humanized monoclonal antibody targets VEGF and is already incorporated in the treatment of colorectal, breast, lung, and kidney cancer. 

The first published trial with bevacizumab in recurrent glioma was a single-arm phase II trial of bevacizumab plus irinotecan that showed the encouraging results of 63% radiologic response, 6-month PFS of 38%, and median PFS of 23 weeks [20]. No CNS haemorrhages were reported, but there were some thromboembolic events. This study was followed by several others, trying to confirm the initial results. In a subsequent phase II trial of single-agent bevacizumab for recurrent GBM, that included forty-eight patients, a radiographic response of 35% and a 6-month PFS of 29% were reported [21]. An important clinical effect of bevacizumab seen in this trial was the decrease of peritumoral edema that led to reductions in the required corticosteroid doses for a significant proportion of the responding patients, even including some that did not meet the PFS6 landmark. This observation has also been confirmed in other bevacizumab-treated patients [22] and it seems to be a characteristic property of other potent anti-VEGF agents like cediranib.

 The previous experience with irinotecan in recurrent gliomas had shown little activity of the agent, and this raised the question of the irinotecan contribution to the bevacizumab results [23, 24]. To investigate this, a phase II trial randomized 167 patients with recurrent GBM to either single-agent bevacizumab or bevacizumab with irinotecan. The response rates were 28% and 38%, respectively, and PFS at 6 months was 43% and 50% [25]. Median overall survival was 9.2 and 8.7 months, confirming again the minimal impact of irinotecan to the results. Following these publications, the FDA granted accelerated approval for the use of bevacizumab in recurrent disease, despite the lack of any randomized data comparing bevacizumab monotherapy or combination against a standard chemotherapy regimen. Recently, the first data of bevacizumab in the adjuvant setting have become available [26]. In this phase II study by Lai et al. seventy patients with newly diagnosed glioma were treated with standard postoperative chemoradiotherapy plus biweekly bevacizumab. A control cohort of newly diagnosed patients treated with first-line RT and TMZ who had mostly received BV at recurrence at the University of California, Los Angeles/Kaiser Permanente was selected for comparison. The overall survival (OS) and progression-free survival (PFS) were 19.6 and 13.6 months, respectively, compared to 21.1 and 7.6 months in the comparison group, showing an improvement in the PFS but not in overall survival. Subgroup analysis data indicate a potential benefit of upfront bevacizumab use for the poor prognosis patients, emphasizing the need for further clinical investigation to identify the optimal use setting.

## 4. Cediranib

Cediranib (AZD2171, Recentin) is an oral pan-VEGFR tyrosine kinase inhibitor with additional activity against platelet-derived growth factor *β* and c-Kit. It has a half-life of 22 hours thus allowing once-daily dosing [27]. 

Based on preliminary data showing a potential benefit in patients with recurrent glioblastoma by normalization of vasculature and alleviation of edema, a phase II study was conducted including 31 patients with recurrent glioblastoma [28]. They were all treated with cediranib 45 mg/d p.o., and the primary endpoint was 6-month progression-free survival. PFS-6 was 25.8%, and treatment was well tolerated. Grade 3-4 toxicities included diarrhea, hypertension, and fatigue. About half of the patients had entered the study while on steroids, and the majority managed to reduce dose or even discontinue their medication while on cediranib. Changes in plasma placental growth factor, basic fibroblast growth factor, matrix metalloproteinase (MMP)-2, soluble VEGF receptor 1, stromal cell-derived factor-1*α*, and soluble Tek/Tie2 receptor and in urinary MMP-9/neutrophil gelatinase-associated lipocalin activity after cediranib were associated with radiographic response or survival, but their value as predictive markers will require further exploring. Disease progression during ongoing treatment with cediranib correlated with increases in bFGF, SDF1-alpha, and viable circulating endothelial cells (CEC). Tumor progression after drug cessation, in contrast, correlated with increase in the number of circulating progenitor cells (CPC), suggesting an independent role of CEC and CPC as biomarkers of treatment response in patients treated with cediranib.

These promising results led to the design of a multicenter three-arm phase III study (REGAL; NCT00777153) with 325 patients, where cediranib alone and combination with lomustine were tested versus lomustine alone in patients with recurrent glioblastoma [29]. PFS at 6 months (PFS-6) was 16% in the monotherapy arm compared with 34.5% in the combination arm and 24.5% in the control arm, and no significant difference has been identified. Therefore, cediranib alone or the addition of cediranib to lomustine did not improve PFS in comparison to lomustine alone. The PFS-6 of 16% in the cediranib arm was lower than the PFS-6 of 25.8% reported in the previous phase II trial. Possibly, the different doses of cediranib used in the two trials, that is, 45 mg in the phase II versus 30 mg in the monotherapy and 20 mg in the combination arm of the phase III trial, may indicate a dose-response property which could be the explanation for the difference in the PFS-6 seen in the two studies.

Currently cediranib is under investigation in paediatric patients with recurrent CNS tumors and in several adult studies. There is an ongoing phase I trial of the combination with gamma-secretase/Notch signalling pathway inhibitor RO4929097 in solid tumors. The addition of gefitinib versus placebo to cediranib is explored in a randomized phase II study in recurrent glioma, while another phase II is evaluating the addition of cediranib versus placebo to chemoradiotherapy in newly diagnosed glioblastoma. Cediranib plus the triple angiokinase inhibitor BIBF1120 in recurrent glioblastoma is tested in a phase II safety and efficacy study and finally cediranib and cilengitide, which targets the *ανβ*3 and *ανβ*5 integrin receptors are combined in a phase Ib study in a recurrent glioblastoma patient population [30].

## 5. Cilengitide (EMD 121974)

Cilengitide is a cyclized arginine-glycine-aspartic acid-containing pentapeptide that selectively binds the cell surface receptors *ανβ*_3_ and *ανβ*_5_, which are expressed on activated endothelial cells during angiogenesis [31]. It can also act directly on *ανβ*_3_- and *ανβ*_5_-expressing tumor cells and inhibit important signals involved in survival and growth and indirectly by inhibiting angiogenesis and thereby tumor growth [32]. In glioblastoma, both activated endothelial cells and tumor cells express the target integrins of cilengitide [33]. Cilengitide has been the first integrin-receptor antagonist to enter clinical development. Data from phase I studies have shown activity in recurrent glioblastoma [34], and a phase II study where 81 patients were randomized to single-agent cilengitide 500 mg or 2000 mg iv. twice weekly until progression has offered evidence of good tolerance and modest activity in both arms, with a trend favoring the 2000 mg dose. PFS-6 was 15%, and OS was 9.9 months [35]. These results together with preclinical data supporting a synergistic effect between cilengitide and radiotherapy [36] provided the rationale to combine this agent with chemoradiotherapy in newly diagnosed glioblastoma. In this phase I/IIa study, 52 patients were treated with cilengitide 500 mg iv. twice weekly in addition to standard chemoradiotherapy with temozolomide until progression or up to 35 weeks [37]. Primary endpoint was 6-month PFS. Six- and 12-month PFS rates were 69% and 33%, and median PFS and OS were 8 and 16.1 months, respectively. PFS and OS were longer in patients with tumors with O6-methylguanine-DNA methyltransferase (MGMT) promoter methylation (13.4 and 23.2 months) versus those without MGMT promoter methylation (3.4 and 13.1 months). The reasons for this finding remain unclear. No synergistic effects were identified for the combination of cilengitide and TMZ in vitro, and the MGMT status had no effect on the biologic activity of cilengitide. A possible explanation could be that the cilengitide-induced vascular normalization improves the delivery of TMZ to tumor tissue, especially in patients with MGMT promoter methylation [31]. 

Currently, cilengitide is being evaluated in a recently completed pivotal, randomized Phase III study (Cilengitide in Combination with Temozolomide and Radiotherapy in Newly Diagnosed Glioblastoma Phase III Randomized Clinical Trial (CENTRIC)) for newly diagnosed Glioblastoma Multiforme with MGMT promoter methylation [38]. Following this trial, a similar one has been designed for patients with unmethylated tumors [39] and their results are eagerly awaited. The combination of cilengitide with sunitinib malate is tested in a pilot biomarker study and chemoradiotherapy with cilengitide or cetuximab are being investigated in a randomized, noncomparative trial in patients with newly diagnosed MGMT-promoter unmethylated glioblastoma (CeCil) [40].

## 6. Sunitinib

Sunitinib is a multityrosine kinase inhibitor which targets PDGFR, vascular endothelial growth factor receptor-2 (VEGFR-2), and the proto-oncogenes RET, KIT, FLT3, and CSF-1. It is FDA-approved as first-line treatment for advanced renal cell carcinoma, hepatocellular carcinoma, and progressive gastrointestinal stromal tumors (GIST) resistant to imatinib. It has also been reported to have activity in renal cell carcinoma CNS metastases [41]. The implication that sunitinib may cross the blood-brain barrier has provided the basis for designing trials to explore its activity in brain tumors. 

An in vitro study in glioma cell lines showed some interesting results regarding the effect of sunitinib in response to temozolomide and radiotherapy. They have demonstrated that adding sunitinib to chemoradiotherapy increased sensitivity of the O(6)-methylguanine-methyltransferase- (MGMT-) positive cells, but did not affect the MGMT-negative ones. MGMT-positive cells displayed higher levels of vascular endothelial growth factor receptor 1 (VEGFR-1) whereas the negative ones displayed decreased levels of VEGFR-2. Also, they showed that MGMT expression was associated with a significant increase in the soluble VEGFR-1/VEGFA ratio, thereby suggesting a decrease in bioactive VEGFA and a shift towards an antiangiogenic profile. This direct correlation between MGMT and reduced angiogenicity and tumorigenicity may suggest a role for sunitinib in combination with standard treatment in MGMT-positive gliomas [42].

In another preclinical study in a mouse tumor model, sequential administration of sunitinib and temozolomide suggested a dose-dependent action of sunitinib on tumor penetration of temozolomide [43].

However, the available clinical data have been less optimistic and sunitinib has not yet demonstrated significant activity in the published trials. In a phase II study, it was given as single agent on recurrence following chemoradiotherapy at a dose of 37.5 mg/day and MRI was used to evaluate the antiangiogenic effects of sunitinib. There were no objective responses, and median time to progression and overall survival were 1.6 and 3.8 months, respectively [44]. 

The combination of sunitinib and irinotecan in a phase I study, where the MTD for sunitinib was 50 mg/day for 4 weeks on a 6-week cycle with Irinotecan at 75 mg/m^2^ every other week, had moderate toxicity but minimal activity [45]. Currently sunitinib is being tested in two phase II trials, one for recurrent anaplastic astrocytoma and glioblastoma and one for inoperable newly diagnosed glioblastoma before and during radiotherapy [46].

## 7. Sorafenib

Sorafenib is an oral VEGFR-2, Raf, PDGFR, c-KIT, and Flt-3 inhibitor currently approved for renal cell and hepatocellular carcinomas, which is also under evaluation for lung and breast cancer. Preclinical data suggest that sorafenib inhibits STAT-3 signalling and therefore contributes to growth arrest and induction of apoptosis in glioblastoma cells. These findings could provide a rationale for potential treatment of malignant gliomas with Sorafenib [47]. 

The addition of Sorafenib 400 mg twice daily to temozolomide in the maintenance period of treatment in newly diagnosed glioblastoma following standard chemoradiotherapy was investigated in a phase II study with 47 patients. 19 withdrew before initiation of maintenance treatment due to progression and only 9 received 6 months of treatment as planned. Median PFS was 6 months, and 1-year PFS was 16%. Median overall survival was 12 months. Maintenance treatment with sorafenib was well tolerated with no grade 3/4 toxicities, but it did not seem to add to the standard treatment effect [48].

Sorafenib is being evaluated in a phase I/II trial in combination with temsirolimus in recurrent glioblastoma and in a phase I trial in combination with standard chemoradiotherapy and maintenance temozolomide in newly diagnosed patients [49].

## 8. Vandetanib

Vandetanib (ZD 6474) is a VEGF and EGF receptor tyrosine kinase inhibitor. The VEGF receptors, VEGFR-1 (flt-1) and VEGFR-2 (KDR), are commonly present on endothelial cells and have also been identified in human glioblastoma cells. In addition, EGFR is dysregulated in the majority of human glioblastomas and EGFR overexpression correlates with shorter survival. The effect of vandetanib as a dual inhibitor of VEGF and EGFR signaling has been studied as single agent and in combination with radiation. Preclinical data have shown that irradiation of glioblastoma xenografts in combination with vandetanib significantly increased tumor doubling time compared with RT alone. The growth delay correlated with suppression of pAkt, survivin, and Ki67 expression in tumor samples. The presence of EGFR augmented RT-stimulated VEGF release; this effect was inhibited by vandetanib. In this study that also included cediranib, neither TKI affected clonogenic cell survival following RT nor GBM xenografts expressing EGFR exhibited greater sensitivity to both cediranib and vandetanib than EGFR-null tumors [50]. In a similar setting, Damiano et al. have shown that when irradiation and vandetanib are applied to glioma xenografts and cell lines, they cause a longer-lasting inhibition of tumor growth and vessel formation, as compared to the reversible inhibition produced by each compound given separately [51].

 Another in vitro study evaluated the effect of vandetanib on the blood-brain barrier (BBB) and the potential detrimental effect of that on chemotherapy activity. They have used glioblastoma xenografts in mice, that were treated with vandetanib, temozolomide, or both. Vandetanib selectively inhibited angiogenic growth aspects of glioma and restored the BBB. It did not notably affect diffuse infiltrative growth and survival. Also, vandetanib antagonized the effects of temozolomide, presumably by restoration of the BBB and obstruction of chemodistribution to tumor cells [52]. These results generate a whole set of questions regarding the efficacy of the concomitant use of antiangiogenic agents with chemotherapy in brain tumors. 

Vandetanib was tested in a phase I study in combination with temozolomide and RT in newly diagnosed glioblastoma. The maximum dose administered was 200 mg daily and the toxicities included gastrointestinal bleeding and perforation, neutropenia, and thrombocytopenia [53]. Vandetanib is now evaluated in an ongoing phase II study, where it is given with temozolomide and RT at the dose of 100 mg daily.

## 9. ABT-510/Thrombospondin-1 (TSP-1)

TSP-1 is a 450-kD homotrimeric extracellular matrix glycoprotein. It has a complex structure and modulates cellular motility, adhesion, and proliferation, functions that are all important for tumor growth and metastasis [54]. TSP-1 has been shown to inhibit angiogenesis by inhibiting endothelial cell proliferation, migration, and cord formation, both in vitro and in vivo [55, 56]. Both intact TSP-1 and derived peptides have been shown to induce apoptosis in endothelial cells [57]. The in vitro antiangiogenic activity of TSP-1 is mediated by the CD36 receptor expressed on endothelial cells [58]. TSP-1 production by glioma cells is reduced under hypoxic conditions, while VEGF is increased, causing an imbalance that favors angiogenesis. Tenan et al. showed that increased expression of TSP-1 by two- to 28-fold suppresses tumorigenicity of glioblastoma cells in an animal model, which indicates that even a modest reduction in TSP-1 production might be relevant to human tumor progression [59]. In a very interesting in vitro study, Filleur et al. demonstrated a potential explanation on how tumors can overcome TSP-1 activity and restore angiogenesis despite high levels of TSP-1 expression, thus developing acquired resistance to antiangiogenic drugs [60].

ABT-510 is a TSP-1 mimetic antiangiogenic drug that has been tested in newly diagnosed glioblastoma on a phase I trial concurrently with temozolomide and RT. Subcutaneous injections of 20–200 mg were used and there were no dose-limiting toxicities identified. The study included gene expression analysis, using TaqMan low-density arrays to identify angiogenic genes that were differentially expressed in the brains of controls, compared with patients with newly diagnosed glioblastoma. FGF-1 and TIE-1 genes were found to be downregulated in patients who had better clinical outcomes [61].

## 10. XL184

XL-184 (BMS-907351) is a pan-tyrosine kinase inhibitor for the potential oral treatment of medullary thyroid cancer, glioblastoma multiforme, and NSCLC. The principal targets of XL-184 are MET, VEGFR-2, and RET, but the drug is also reported to display inhibitory activity against KIT, FLT3, and TEK. Also, it is known that elevated MEK and KIT correlate with poor prognosis. Preclinical studies demonstrated that XL-184 potently inhibited multiple receptor tyrosine kinases in various cancer cell lines and animal xenograft models. Phase I data indicated that XL-184 accumulated dose dependently in the plasma and had a long terminal half-life. Results from a phase II study in glioblastoma on first or second relapse showed that the drug is active and can produce responses at the dose of 175 mg PO qd, in a group of patients who had previously been treated with antiangiogenic agents. However, toxicity had been an issue in this trial, as 52% of the 46 enrolled patients interrupted or stopped treatment because of an adverse event [62]. These included myocarditis, troponin elevation, pulmonary embolism, CNS hemorrhage, nausea, fatigue, and dehydration. A second cohort was added subsequently, that received a dose of 125 mg. In the 175 mg qd cohort (*n* = 46), PFS6 (all pts) was 21% and median duration of response was 5.9 months. Most frequent Gr3/4 AEs were fatigue (23%), hypophosphatemia (10%), serum lipase elevation (10%) and ALT, headache, lymphopenia, and convulsion (9% each), with similar effect on both dose levels [63]. Currently, there are two ongoing phase II trials with XL184, studying the administration as single agent in astrocytoma and glioblastoma, respectively and a phase I safety study that evaluates incorporation of XL184 in the standard chemoradiation program for newly diagnosed glioblastoma, giant cell glioblastoma, and gliosarcoma [64]. 

## 11. VEGF Trap/Aflibercept

VEGF Trap/aflibercept has been developed by incorporating domains of both VEGF receptor 1 (VEGFR-1) and VEGFR-2 fused to the constant region of human immunoglobulin G1, which acts as a soluble decoy receptor for VEGF. It is known to have very high affinity for all isoforms of VEGF-A (<1 pM), as well as placental growth factor, a closely related angiogenic factor [65]. Its efficacy has been proven in several preclinical studies of solid tumors and in a subcutaneous glioma model [66–68]. In a preclinical study with animals bearing intracranial glioma xenografts, it produced improved survival when given in a prolonged schedule and maintained its efficacy both in early-stage and advanced disease [69]. Until now, there has been one phase II trial in glioma at first relapse that is pending publication and a new phase I trial not opened yet, where VEGF Trap is applied together with standard chemoradiotherapy with temozolomide in untreated patients [70]. 

## 12. Tandutinib

Tandutinib (MLN518) is a 4-piperazinyl quinazoline compound that is a potent inhibitor of type III receptor tyrosine kinases (RTKs) including PDGFR-*β*, FLT-3, and c-Kit. It has been extensively studied in the context of acute myeloid leukemia in combination with approved drugs and showed significant activity and synergistic properties [71]. It has been tested in glioma patients in a feasibility and phase I study at doses from 500 to 700 mg bid. The observed toxicities included fatigue, somnolence, and phosphorus disorders, and the recommended dose for phase II was set at 600 mg. There are currently two ongoing tandutinib studies in glioma, one phase II in relapsed disease in combination with bevacizumab and one feasibility and phase I/II trial in relapse after radiotherapy with or without chemotherapy, to establish maximum dose and pharmacokinetic profile [72].

## 13. Conclusions

Management of glioblastoma remains a challenging area in oncology. The current approved treatments offer survival rates that are far from satisfactory, and clinical research is intensively trying to identify more effective agents to incorporate in clinical use. The particular pathologic and molecular features that characterize glioblastoma and regulate its clinical course are currently investigated in conjunction with new targeted agents which, if proven efficient, might give the answers so eagerly awaited. Angiogenesis undoubtedly plays a critical role in the development and survival of glioblastoma; it is therefore expected that the antiangiogenic drugs will remain key players in the treatment design. It is questionable, though, whether we have sufficiently understood their effects in the tumor environment, their interaction with chemotherapy agents, and their mode of application that can lead to optimum results.
